# Updated solution for diagnosis and management of calcinosis cutis: A retrospective review

**DOI:** 10.1097/MD.0000000000039139

**Published:** 2024-08-09

**Authors:** Ki Hyun Kim, Kyung Min Kim, Sang Seok Woo, Se Ho Shin, Jai Koo Choi, Seong Hwan Kim, Jun Won Lee, In Suck Suh

**Affiliations:** a Department of Plastic and Reconstructive Surgery, Kangnam Sacred Heart Hospital, College of Medicine, Hallym University, Seoul, Republic of Korea.

**Keywords:** calcinosis cutis, calcium crystals, imaging, wound healing

## Abstract

Calcinosis cutis is classified into 5 main types: dystrophic, metastatic, idiopathic, iatrogenic, and calciphylaxis. However, it is occasionally misdiagnosed as a malignancy and its management remains challenging. Therefore, in this study, we report our diagnostic and treatment experiences with patients with calcinosis cutis and suggest strategies for improving patient care. This retrospective study included 7 patients (4 men, 3 women; 44.4 ± 32.0 years old) who visited our hospital between March 2013 and December 2022 and were diagnosed with calcinosis cutis through histopathological procedures. The patients underwent complete excision of the mass without a safety margin. Frozen biopsy was not performed during surgery. No significant intraoperative or postoperative complications were noted after the application of various imaging techniques for diagnosis and follow-up. All patients showed complete recovery. Follow-up showed no recurrence or complications in the 6 patients who completed 1 year of follow-up. Radiological tests such as plain radiography, ultrasonography, computed tomography, and magnetic resonance imaging are important for accurate diagnosis and treatment of calcinosis cutis. This approach can ensure precise assessment of preoperative lesions, leading to safe and less invasive patient treatment, recurrence prevention, and complications of calcinosis cutis.

## 1. Introduction

Calcinosis cutis is a condition in which calcium salts are deposited in the skin and subcutaneous tissue. It is classified into 5 main types: dystrophic, metastatic, idiopathic, iatrogenic, and calciphylaxis.^[1]^ Dystrophic calcinosis cutis is the most common type of cutaneous calcification.^[2]^ Despite extensive research, the pathophysiology of calcinosis cutis remains elusive, making diagnosis challenging, particularly in the absence of associated symptoms such as ulcers or other comorbidities.^[3]^

To diagnose calcinosis cutis, the patient’s medical history and laboratory findings are evaluated to examine associations with connective tissue diseases or metabolic imbalances.^[4]^ Usually, imaging studies, such as plain radiography, ultrasound, computed tomography (CT), and magnetic resonance imaging (MRI), can be valuable for identifying the location and extent of calcium deposits.^[4]^ However, while physical examination and radiological findings can provide localization information, a definitive diagnosis necessitates a biopsy and subsequent histopathological examination.^[4]^

Given the lack of a standard method for diagnosing calcinosis cutis prior to surgery, clinical diagnosis of calcinosis cutis is often achieved through time-consuming histopathological or immunohistochemical procedures.^[5]^ Consequently, this condition is frequently misdiagnosed as skin conditions such as pilomatrixoma, juvenile xanthogranuloma, molluscum contagiosum, and malignancy.^[6]^ Several therapeutic approaches have demonstrated varying degrees of effectiveness in the treatment of calcinosis cutis, including topical and intralesional sodium thiosulfate, colchicine, extracorporeal shockwave lithotripsy, and surgical excision.^[7]^

However, a standard and clear diagnostic and treatment approach has not been established.^[3]^ This makes the management of this condition in clinical fields extremely challenging. The excessive use of diagnostic tests results in significant cost wastage, and performing minimally invasive treatments without complications proves to be a difficult task. Therefore, in this study, we aimed to explore potential diagnostic and treatment methods for calcinosis cutis by reporting our experience with various forms and types of this condition across different parts of the body.

## 2. Materials and methods

Following ethical approval, we performed a retrospective review of 7 patients who were histopathologically diagnosed with calcinosis cutis at our hospital from March 2013 to December 2022. Since there is no established treatment of choice for calcinosis cutis, we chose surgical treatment as the most appropriate option for all patients.

Patient medical and trauma histories were examined before surgery (Table 1). We also conducted preoperative blood tests for assessing serum calcium and phosphate levels and imaging tests such as plain radiography, ultrasound, CT, and MRI (Table 2). The patient group underwent complete excision of the mass without safety margins. Frozen biopsy was not performed during surgery. To cover the defects that occurred after surgery, a local flap was created. Hematoxylin and eosin (H&E) staining was performed for histopathological analysis of the biopsy specimens. Clinical follow-up and imaging tests were performed to assess recrudescence.

**Table 1:** Anthropometric data for the study participants, including age, sex, and any additional relevant information.

Patient number	Sex/age (years)	Past medical history	Onset of calcinosis cutis	Trauma history	Location of calcinosis cutis	Number of lesions
1	M/70	Lymphoma diabetic mellitus dyslipidemia	2 months	+	Lower leg	>10
2	M/20	–	2 years	+	Buttock	4
3	M/55	–	20 years	+	Scrotum	>10
4	F/53	Kyphosis	1 month	+	Back	4
5	M/12	–	3 years	–	Chin	1
6	F/45	–	3 months	–	Breast	>10
7	F/56	–	3 months	–		>10

**Table 2:** Comprehensive summary of the preoperative medical test data for the study participants.

Patient number	Preoperative radiograph	Preoperative laboratory findings: Ca/phosphate levels (8.4–10.2 mg/dL/2.6–4.62 mg/dL)	Type of calcinosis cutis	Postoperative radiography	Complication
1	Ultrasonography MRI plain radiography CT	9.1/3.3	Dystrophic	Plane radiograph ultrasonograph CT	–
2	Ultrasonography CT	9.9/4.0	Dystrophic	Ultrasonograph	–
3	Ultrasonography CT	9.6/3.5	Dystrophic	Ultrasonograph plane radiograph	–
4	Ultrasonography	9.6/4.5	Dystrophic	Ultrasonograph	–
5	Ultrasonography	9.9/4.5	Idiopathic	Ultrasonograph	–
6	CT MRI ultrasonography plain radiography	8.3/3.3	Iatrogenic	Ultrasonograph	–
7	CT ultrasonography plain radiography	7.7/4.1	Iatrogenic	Ultrasonograph	–

CT = computed tomography, MRI = magnetic resonance imaging.

## 3. Results

This study included 7 patients who were histopathologically diagnosed with calcinosis cutis. Patient demographics in our study showed a broad age range, spanning from 12 to 70 years (Table 1). Among them, 4 had a history of frequent bumping or rubbing the lesioned area. Physical examinations revealed hard, fixed, and well-defined masses.

The 7 patients had lesions of varying size and shape in different locations, including the jaw, testicles, back, buttocks, breast, and legs. Six patients had multiple lesions, and the size and shape of these lesions varied significantly (Table 1). Preoperative blood tests revealed that all 7 patients had normal calcium and phosphate levels. Preoperative imaging tests such as ultrasound, plain radiograph, MRI, and CT were performed (Fig. 3). Ultrasound revealed hyperechoic deposits with a posterior acoustic shadowing artifact due to the acoustic properties of calcium (Fig. 3).

Through preoperative physical examination, imaging, and blood tests, we identified 1 patient with the idiopathic type, 4 with the dystrophic type, and 2 with the iatrogenic type (Table 2) of calcinosis cutis. All these patients underwent complete excision of the mass without including a safety margin, regardless of the type. They underwent surgery under either local or general anesthesia depending on the size, location, and number of lesions.

**Figure 1.**
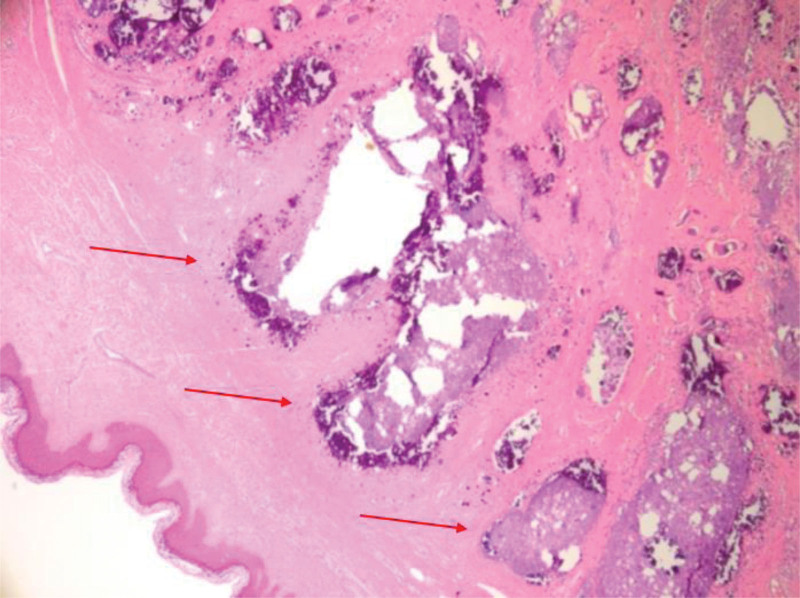
Hematoxylin and eosin (H&E) staining of the biopsy specimens (H&E, × 400). Irregular deposits of intensely basophilic acellular material in the dermis and subcutaneous tissue.

To cover the defects that occurred after surgery, a local flap was created. Through preoperative imaging tests, the depth and size of the lesion were predicted, resulting in minimal bleeding with no intraoperative complications. All 7 patients had calcium deposits within the dermis, which sometimes extended into the subcutaneous tissue, as seen in their tissue biopsies (Fig. 1). These deposits appeared as basophilic (blue) granules or amorphous materials upon H&E staining (Fig. 1). In 2 cases where the mass was associated with a chronic wound, we observed varying degrees of inflammation surrounding the calcium deposits, often accompanied by inflammatory cells such as lymphocytes, histiocytes, and multinucleated giant cells. No significant intraoperative complications were noted, and all patients recovered uneventfully. Follow-up showed no evidence of recrudescence or specific complications such as hypertrophic scars, wound dehiscence, or seroma in 6 patients who completed 1-year follow-up.

### 3.1. Case 1

**Figure 2.**
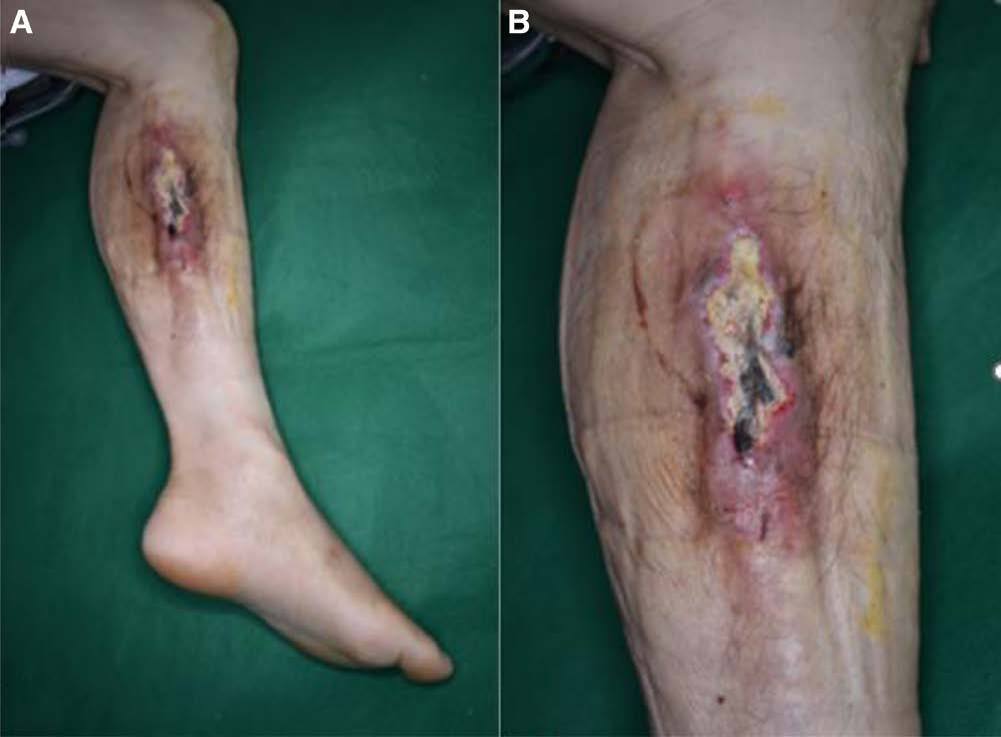
Preoperative images. The mass is accompanied by a 15 × 3 cm-sized chronic ulcer, which is combined with sloughing eschar.

**Figure 3.**
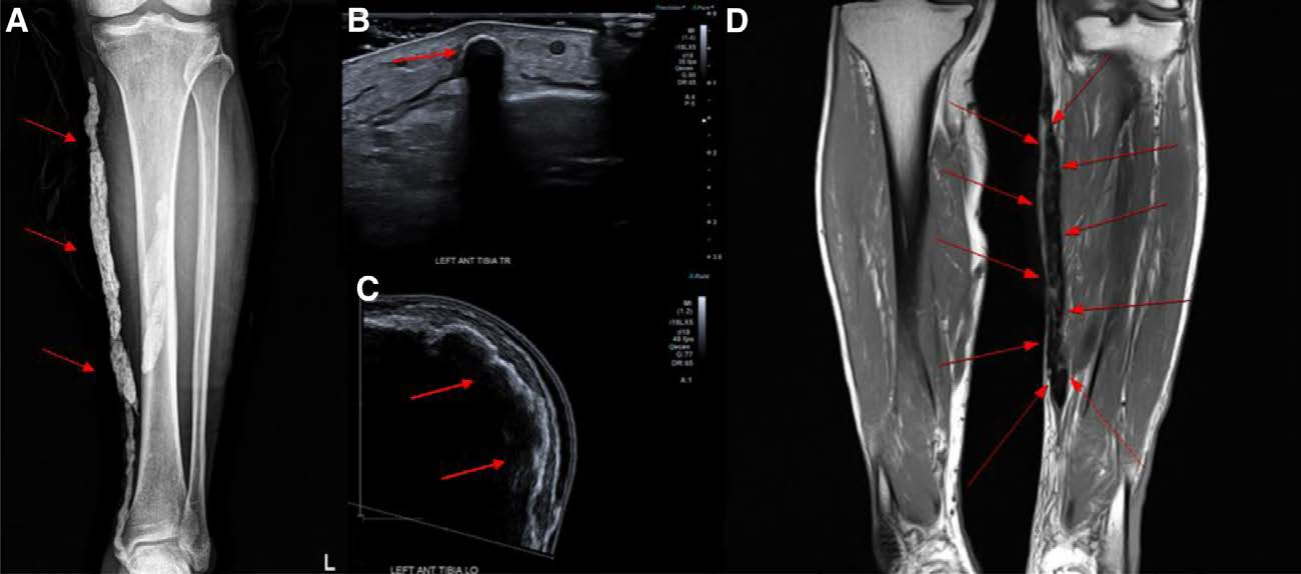
A 70-year-old man with calcinosis cutis. (A) Preoperative plane radiograph showing irregular rod-shaped radiopaque areas measuring 18 cm in length. (B and C) Ultrasonography image showing superficial subcutaneous fat layer with an ill-defined hypoechoic lesion with soft tissue swelling and hyperemia, indicating subcutaneous abscess with cellulitis. (D) Magnetic resonance image showing multifocal, linear, tubular and lobulated dark signal intensity calcified lesion.

**Figure 4.**
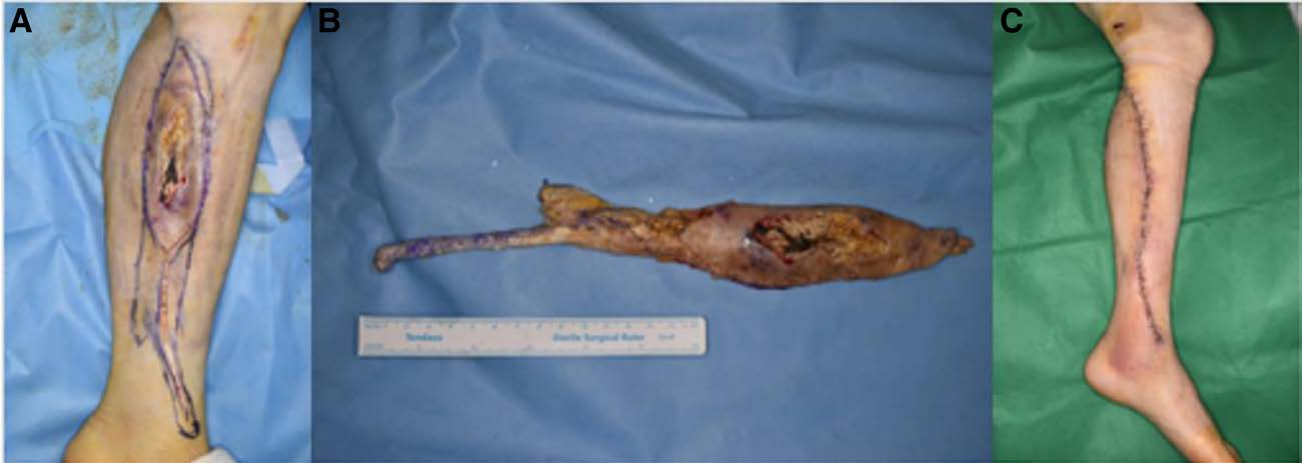
A 70-year-old man with calcinosis cutis. (A) Preoperative design. (B) Resected mass image showing fragment of the skin with soft tissue measuring 27 × 4.5 × 3 cm in size. (C) Postoperative image showing the defect was covered by a local flap.

**Figure 5.**
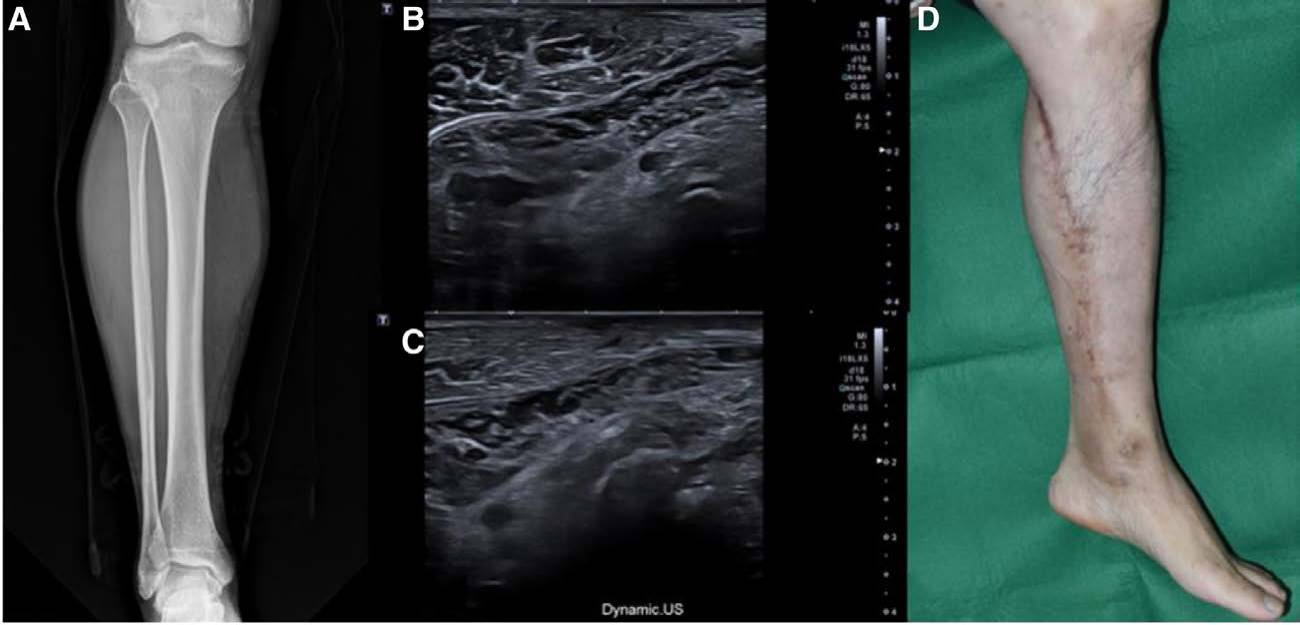
A 70-year-old man with calcinosis cutis. (A) Plane radiograph at the 1-year follow-up. Ultrasonography images showing (B and C) no signs of recurrence. Photo (D).

A 70-year-old man presented to our hospital with a chronic ulcerative wound on the left anterior calf (Fig. 2). His medical history included a diagnosis of lymphoma 30 years earlier, for which he had undergone chemotherapy. He also had cellulitis in the calf, for which he received intravenous chemotherapy. Recently, he had sustained an injury to the same calf by falling onto a cement floor. On examination, a palpable hard mass beneath the wound, larger than the wound itself, raised suspicions of a foreign body. For further investigation, plain radiography, ultrasonography, and MRI of the lower extremities were performed, which showed the presence of multiple radiopaque rod-like materials in the anterior calf (Fig. 3). Subsequently, we performed complete excision of the mass and performed a biopsy by resecting a 26 × 4.5 × 3-cm skin and soft tissue mass. Postoperatively, a local flap was applied (Fig. 4), the flap site healed, and the patient recovered without any complications. H&E staining of the biopsy specimen confirmed the diagnosis of calcinosis cutis. Follow-up assessments, including plain radiographs and ultrasonography, indicated the absence of calcified lesions, and the patient reported no recrudescence or complications during the 1-year follow-up period (Fig. 5).

### 3.2. Case 2

**Figure 6.**
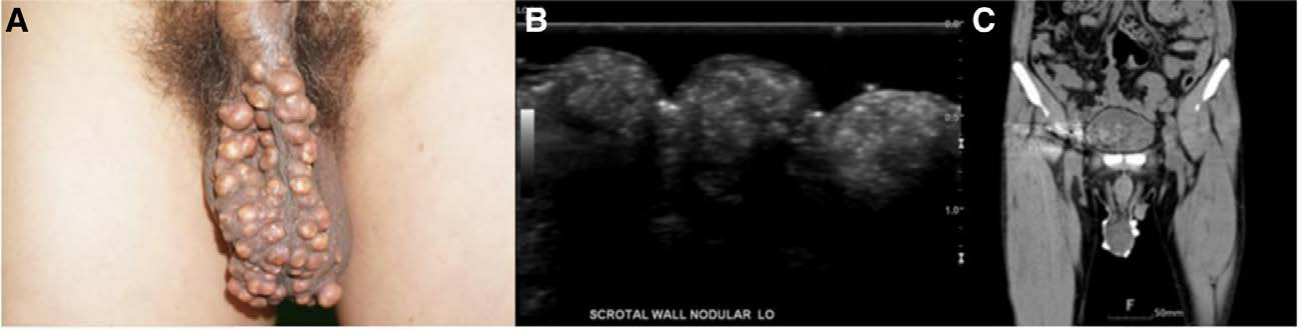
A 55-year-old man with calcinosis cutis. (A) Preoperative image showing multiple nodular masses with a diameter of approximately 1 cm. (B) Ultrasonography image showing multiple round and oval-shaped hyperechoic nodules of variable sizes in along the scrotal wall, without vascularity. (C) Computed tomography image showing multiple round and oval-shaped lesions of variable sizes attenuated along the scrotal wall.

**Figure 7.**
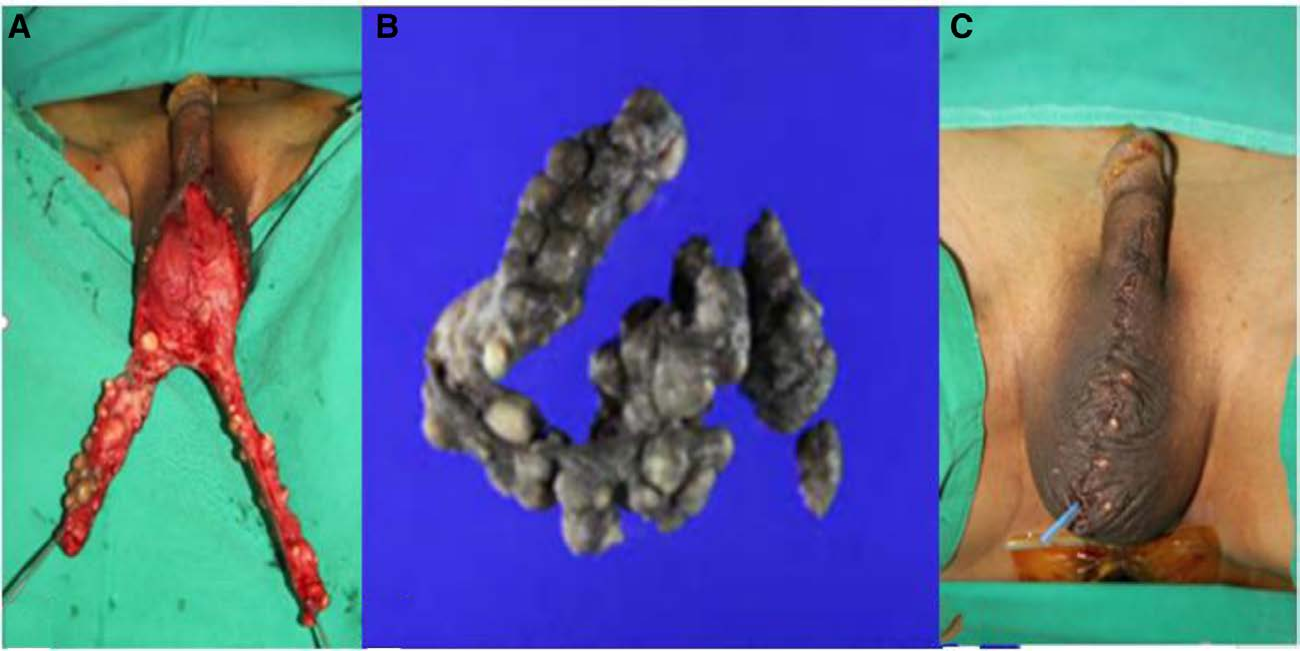
A 55-year-old man with calcinosis cutis.(A) Intraoperative image showing multiple round- to oval-shaped masses with a firm consistency and a yellow color were excised along the scrotal wall. (B) Resected mass image showing several fragments of the skin (up to 21 × 3.3 × 0.5 cm) with multiple calcified nodules (up to 1.5 × 1.2 cm) in the dermis. (C) Postoperative image showing the defect covered by a local flap.

A 55-year-old man presented to our hospital with multiple oval-shaped, hard, and firm masses on the scrotum (Fig. 6). He had no relevant medical history; however, his occupation predominantly involved prolonged periods of sitting, exposing the lesion to continuous pressure and irritation, thus, making it vulnerable. Upon examination, multiple oval-shaped, dome-like hard masses were identified on the scrotum, suggesting an epidermal cyst, pilomatrixoma, or foreign material. Ultrasonography and CT revealed an oval-to-round hyperechoic mass without vascularity along the scrotal wall (Fig. 6). Furthermore, we performed a biopsy by resecting several fragments of skin (21 × 3.3 × 0.5 cm) with multiple calcified nodules (1.5 × 1.2 cm) in the dermis. Following the biopsy, we applied a local flap (Fig. 7), and the flap site healed without complications. Unfortunately, follow-up data for this case were unavailable because he no longer visited the hospital.

## 4. Discussion

Calcinosis cutis is characterized by calcium deposition in subcutaneous soft tissues^[8]^ and can present anywhere in the body as painless nodules or swellings. The extremities and buttocks have been reported as the most frequently affected sites.^[9]^ Calcinosis cutis can be dystrophic, associated with tissue damage, or metastatic, associated with elevated calcium or phosphate levels. The other patients had iatrogenic or idiopathic calcinosis cutis.^[10]^ The idiopathic type occurs without prior tissue damage, while the iatrogenic type usually occurs after intravenous calcium or para-aminosalicylic acid treatment.^[3]^ Calciphylaxis involves the calcification of small and medium-sized vessels and is typically associated with chronic renal failure and dialysis. The disorder is classified as calcinosis circumscripta when it is limited to an extremity or joint.^[1]^ Skin and subcutaneous tissue calcification can also occur in various disorders.^[2]^ Additionally, other neoplasms are associated with skin calcification.^[11]^

The diagnosis of calcinosis cutis is challenging because there is no established diagnostic procedure yet. The patient’s history, radiologic, and laboratory findings can help, but there is a high possibility of misdiagnosis with other conditions such as pilomatrixoma, molluscum contagiosum, juvenile xanthogranuloma, and even neoplasm. This poses difficulties for clinicians in both diagnosis and treatment. Therefore, the management of calcinosis cutis remains challenging.^[6,7]^

In this study, we present our findings while managing calcinosis cutis that manifested in various forms and types across different body parts. Based on our experience, we proposed a standardized method for the accurate diagnosis and appropriate treatment of calcinosis cutis.

Physicians are advised to perform physical examinations and history-taking of patients presenting with skin and soft tissue masses. History-taking should include questions designed to determine how long the mass has been present, and the nature and duration of any associated symptoms. Any changes in the size or growth rate of the mass and any prior history of cancer should be noted.^[12]^ Calcinosis cutis can be accompanied by various comorbidities, such as ulcers or chronic wounds, and is relatively distinct in its region, smooth on the surface, immobile, and firm. In these patients, measuring calcium and phosphate levels is recommended.

Calcinosis cutis can be associated with both normal and elevated calcium levels.^[13]^ Elevated serum Ca2+ and/or Ph4+ levels have been observed in metastatic calcinosis, idiopathic calcinosis, and calciphylaxis, which can be related to other systemic diseases.^[2]^ In our experience, imaging tests such as plain radiography, ultrasonography, CT, and MRI are more crucial in the diagnosis of calcinosis cutis than the previously mentioned diagnostic examinations such as history-taking, physical examinations, and laboratory findings. We recommend performing plain radiography and ultrasonography as the first step. Plain radiography has a limited role in the definitive diagnosis and staging of soft tissue tumors. However, it is very sensitive in detecting calcinosis and allows the assessment of the extent and approximate size of the mass.^[14]^ Meanwhile, ultrasonography specifically presents hyperechoic deposits with a posterior acoustic shadowing artifact, owing to the acoustic properties of calcium.^[4]^ It can provide a more precise assessment of the location, size, and relationship of the mass with the surrounding tissues.

Sufficient information for diagnostic impressions can be obtained using plain radiography and ultrasonography; however, in cases where the mass is sizable and deeply situated, sole reliance on this information may prove insufficient; performing CT or MRI can offer a more accurate understanding of the mass’s size and its relationship with adjacent tissues, thereby facilitating safer treatment options. Upon establishing an impression of calcinosis cutis using the aforementioned diagnostic methods, irrespective of the specific subtype involved, the most highly recommended treatment approach is excision. During surgical excision, complete removal of the mass without a safety margin can lead to successful treatment without recurrence. Depending on the size and location of the resulting defects after removing the calcified tissue, the wound can be treated by primary closure, flap coverage, or a skin graft.^[15]^ Following surgery, a biopsy was performed to establish the final diagnosis.

In terms of patient follow-up, we recommend ultrasonography and plain radiography as the most important tests, as these examinations can clearly identify recurrence and complications. Through this study, we propose that radiological tests are the most crucial tests for the accurate diagnosis of calcinosis cutis. This approach allows for a more precise assessment of preoperative lesions, leading to safer and less invasive treatment as well as prevention of recurrence or complications. Furthermore, radiological tests are important during follow-up to detect relapse and complications.

The findings of this study provide valuable insights into the diagnosis and treatment of calcinosis cutis. The accurate diagnosis and appropriate treatment of this condition can help prevent complications and improve patient outcomes.

This study has some limitations, including its small sample size, retrospective design, loss of patients to follow-up, single-center design, and incomplete data regarding patient characteristics. These limitations may affect the generalizability and validity of the conclusions of this study. Future studies with larger sample sizes, prospective designs, longer follow-up periods, and multicenter settings are required to validate these findings and increase the external validity of the results.

Nonetheless, this study provides valuable insights into the diagnosis and treatment of calcinosis cutis. By highlighting the significance of these findings, this study can help healthcare professionals diagnose and treat the condition more effectively and improve patient outcomes.

## Author contributions

**Supervision:** In Suck Suh.

**Writing – original draft:** Ki Hyun Kim.

**Writing – review & editing:** Ki Hyun Kim, Kyung Min Kim, Sang Seok Woo, Se Ho Shin, Jai Koo Choi, Seong Hwan Kim, Jun Won Lee.
